# In Silico Subtractive Proteomics and Molecular Docking Approaches for the Identification of Novel Inhibitors against *Streptococcus pneumoniae* Strain D39

**DOI:** 10.3390/life13051128

**Published:** 2023-05-04

**Authors:** Ashwag Shami, Nada K. Alharbi, Fatimah A. Al-Saeed, Aiman A. Alsaegh, Khalid M. Al Syaad, Ibrahim H. A. Abd El-Rahim, Yasser Sabry Mostafa, Ahmed Ezzat Ahmed

**Affiliations:** 1 Department of Biology, College of Sciences, Princess Nourah Bint Abdulrahman University, Riyadh 11617, Saudi Arabia; 2 Research Centre, Department of Biology, College of Science, King Khalid University, Abha 61413, Saudi Arabia; 3 Advanced Material Science (RCAMS), King Khalid University, Abha 61413, Saudi Arabia; 4 Department of Laboratory Medicine, Faculty of Applied Medical Sciences, Umm Al-Qura University, Makkah Al-Mukarramah 24382, Saudi Arabia; 5 Biology Department, Faculty of Science, King Khalid University, P.O. Box 9004, Abha 61413, Saudi Arabia; 6 The Research Center, Faculty of Science, King Khalid University, P.O. Box 9004, Abha 61413, Saudi Arabia; 7 Department of Environmental and Health Research, Umm Al-Qura University, P.O. Box 6287, Makkah Al-Mukarramah 21955, Saudi Arabia; 8 Department of Theriogenology, Faculty of Veterinary Medicine, South Valley University, Qena 83523, Egypt

**Keywords:** *Streptococcus pneumoniae*, subtractive proteomics, molecular docking, essential proteins, ADMET analysis

## Abstract

*Streptococcus pneumoniae* is a notorious Gram-positive pathogen present asymptomatically in the nasophayrnx of humans. According to the World Health Organization (W.H.O), pneumococcus causes approximately one million deaths yearly. Antibiotic resistance in *S. pneumoniae* is raising considerable concern around the world. There is an immediate need to address the major issues that have arisen as a result of persistent infections caused by *S. pneumoniae*. In the present study, subtractive proteomics was used in which the entire proteome of the pathogen consisting of 1947 proteins is effectively decreased to a finite number of possible targets. Various kinds of bioinformatics tools and software were applied for the discovery of novel inhibitors. The CD-HIT analysis revealed 1887 non-redundant sequences from the entire proteome. These non-redundant proteins were submitted to the BLASTp against the human proteome and 1423 proteins were screened as non-homologous. Further, databases of essential genes (DEGG) and J browser identified almost 171 essential proteins. Moreover, non-homologous, essential proteins were subjected in KEGG Pathway Database which shortlisted six unique proteins. In addition, the subcellular localization of these unique proteins was checked and cytoplasmic proteins were chosen for the druggability analysis, which resulted in three proteins, namely DNA binding response regulator (SPD_1085), UDP-N-acetylmuramate—L-alanine Ligase (SPD_1349) and RNA polymerase sigma factor (SPD_0958), which can act as a promising potent drug candidate to limit the toxicity caused by *S. pneumoniae*. The 3D structures of these proteins were predicted by Swiss Model, utilizing the homology modeling approach. Later, molecular docking by PyRx software 0.8 version was used to screen a library of phytochemicals retrieved from PubChem and ZINC databases and already approved drugs from DrugBank database against novel druggable targets to check their binding affinity with receptor proteins. The top two molecules from each receptor protein were selected based on the binding affinity, RMSD value, and the highest conformation. Finally, the absorption, distribution, metabolism, excretion, and toxicity (ADMET) analyses were carried out by utilizing the SWISS ADME and Protox tools. This research supported the discovery of cost-effective drugs against *S. pneumoniae*. However, more in vivo/in vitro research should be conducted on these targets to investigate their pharmacological efficacy and their function as efficient inhibitors.

## 1. Introduction

*Streptococcus pneumoniae*, commonly referred to as pneumococcus, is a type of Gram-positive bacteria and is generally found in the upper respiratory tract of humans, which is the prime origin of death and morbidity worldwide. *S. pneumoniae* can become pathogenic under various conditions and cause several diseases such as pneumonia, osteomyelitis, bacteremia, otitis media (OM), and sinusitis [1]. According to the WHO, in Pakistan, 92,000 children die due to pneumonia every year, making it a leading killer of children under five years [2]. In 2013, 2.6 million deaths occurred worldwide due to lower respiratory infections, while in 2015, the ratio tremendously increased to 2.74 million [3]. Since the 1980s, however, a dramatic rise in antibiotic resistance within *S. pneumoniae* has been observed in various regions of the globe. Despite the presence of antibiotics and conjugate vaccines, pneumococcus is the most prevalent cause of bacterial OM and is considered the leading cause of pre-clinical visits and antibiotic treatment in the United States. However, the widespread prevalence of resistant strains creates treatment issues. Notably, Penicillin resistance affects over 40% of strains and frequently promotes resistance to subsequent antibiotics namely macrolides and tetracycline, making it an alarming situation globally [4]. In addition, the prevalence of the penicillin-impervious pneumococcus strain found in the children’s upper respiratory tract remains above 40% in the United States [5]. Hence, increased antibiotic resistance against *S. pneumoniae* is a significant concern worldwide [6]. Moreover, *S. pneumoniae* is pervasive, and competence-dependent employing horizontal gene transfer makes it far easier for pathogenicity and antimicrobial resistance determinants to spread throughout the strains [7]. To develop effective prevention and treatment protocols for *S. pneumoniae*, serotype surveillance and knowledge of the incidence of drug-resistant strains in the wider population are essential [8].

Streptococci D39 is a serotype-2 strain that was discovered to have an unexpectedly high risk of causing meningitis and has been the most common serotype-specific responsible for childhood meningitis in developing countries over the past decade. Persistent illness incidence or progressive dispersal would pose a significant potential infection risk since serotype-2 is not included in Polysaccharide Conjugate Vaccines (PCVs) that are currently licensed or in development [9]. Notably, D39 remains incredibly virulent in murine infection modes and is probably the strain employed in most recent studies [10]. The alarming increase of drug-resistance strains in many diseases caused by bacteria, such as *S. pneumoniae*, has created a tremendous need for finding an efficient treatment because current drugs are ineffective, resulting in treatment failure [11]. Unfortunately, microorganisms frequently develop antibiotic resistance, making them difficult to control [12]. Rapid efforts are required to design a therapeutic approach to combat this disease early, considering the risk of antiseptic immunity developing after a while and the harm produced via the organism.

The latest research involves the utilization of an in silico subtractive proteomics approach to recognize and identify new and potent drug targets in the *S. pneumoniae* D39 strain. The present study encloses high throughput screening of *S. pneumoniae* strain D39 to find a non-homolog essential gene involved in a unique metabolic pathway to find potent drug targets. This approach is applied to the genome of a particular pathogen and subtracts the genome until essential genes are found. Subtraction of the pathogenic genome includes the removal of paralogs, removal of host homologous sequences, and identification of essential genes among non-homologous sequences. Now, these unique and essential proteins of the pathogen are subjected to pathway analysis to understand their involvement in the unique metabolic pathways of that pathogen. Druggable targets are identified against those unique pathways. Finally, molecular docking of drug targets was performed with FDA-approved drugs and phytochemicals to check the interaction and binding affinity between predicted drug targets and ligands.

## 2. Methodology

**Figure 1:**
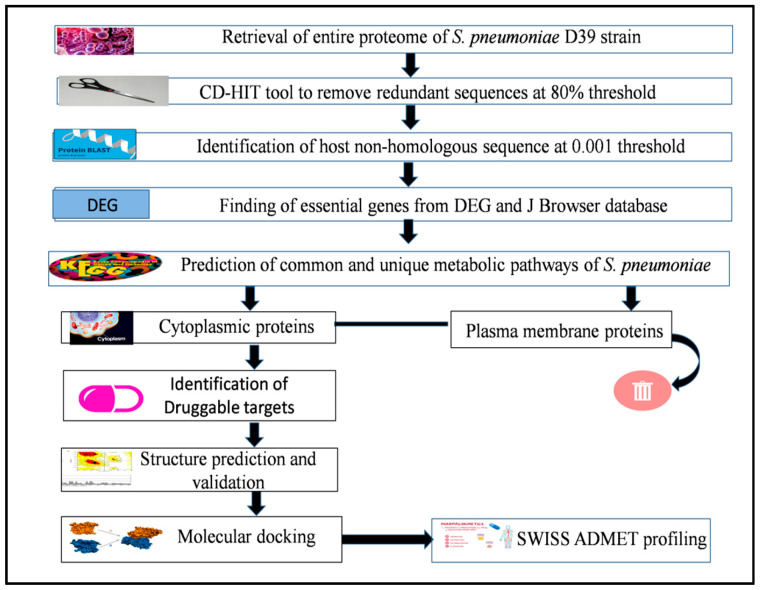
Flowchart showing the overall scheme of the work for detecting drug targets in *S. pneumoniae* strains D39.

The whole proteome of *Streptococcus pneumoniae* strain D39 was used in the present study to identify the novel drug targets. The steps involved in identifying novel drug targets in the *S. pneumoniae* D39 strain are illustrated in Figure 1.

### 2.1. Retrieval of Proteome

The entire proteome of *S. pneumoniae* strain D39 was downloaded from a UniProt database in FASTA format. UniProt is considered the most reliable and accurate database [13].

### 2.2. Removal of Redundant Sequences

A CD-HIT analysis was conducted to exclude duplicated sequences from the proteome [14] because redundant proteins are not required, only a single copy of each protein is required for optimal function. A percentage criterion of 80% has been imposed in the parameters [15].

### 2.3. Elimination of Homologous Protein Sequences

Non-redundant proteins were subjected to BLASTp against Reference Sequence *Homo sapiens* (Tax ID: 9606) by selecting the threshold of 0.001 to eliminate homologs proteins [16] because they generate autoimmune responses. Notably, the proteins exhibiting similarity occurred in “Hits” considered as homologous sequences present among pathogen and host. In addition, non-homologous sequences exhibiting “No-Hits” were considered for further analysis.

### 2.4. Determination of Essential Proteins of Pathogen

Essential genes are required for the daily functioning of cells, such as in protein production, multiplication, cell division, metabolism, etc., and are necessary for the pathogen’s survival [17]. The data on essential genes were gathered from the DEG database with the threshold of 10^−5^ and results were further cross checked by J Browse [18]. Similarly, Pneumo Browse is a graphical and user-friendly interface for exploring the genomic and transcriptome landscape of strain D39V of *S. pneumoniae* [19].

### 2.5. Finding of Metabolic Pathways in S. pneumoniae

This step assisted us in identifying the distinct pathways that were not present in the host by utilizing the KEGG database so that the host’s pathways are not disrupted or altered due to a drug. Only unique pathways to the bacteria were considered to avoid cross-reactivity. Thus, proteins with unique metabolic pathways were chosen for additional research [20].

### 2.6. Sub-Cellular Localization of Essential Proteins

It is significant to predict the function of a specific protein in order to recognize viable therapeutic targets for that protein. Their sub-cellular localization determines the proper functioning of specific proteins. For this purpose, an online localization tool called PSORTb [21] was utilized and the results were further cross-checked by the CELLO tool [22]. Due to protein localization at multiple places, investigations have revealed that localization is a critical dimension for designing any therapeutic options.

### 2.7. Functional Classification of Essential Hypothetical Proteins

Functional family prediction is necessary for identifying functional classes of hypothetical essential proteins. For this purpose, InterProScan was utilized for the functional family prediction of separated imaginary protein sequences. For a protein to be druggable, its functional family must be recognized so that we can quickly determine the targeted protein’s role when designing a drug for it [23].

### 2.8. Identification of Druggable Targets

Finally, a druggability investigation of screened cytoplasmic proteins was carried out with the help of the DrugBank database. Drug Bank contains clinical information about drugs, their side effects, their interactions, etc. [24].

### 2.9. Structure Prediction and Validation

The PDB was searched for the structures of selected proteins Blastp against the PDB was used to acquire an appropriate template for protein structure with the help of Swiss modeling. It led to selecting proteins with various query coverage and percentage similarity. Additionally, four tools (Verify3D, ERRAT, Procheck, and ProSA-web) were used to assess the quality of predicted structures [25].

### 2.10. Molecular Docking and Visualization

**Figure 2:**
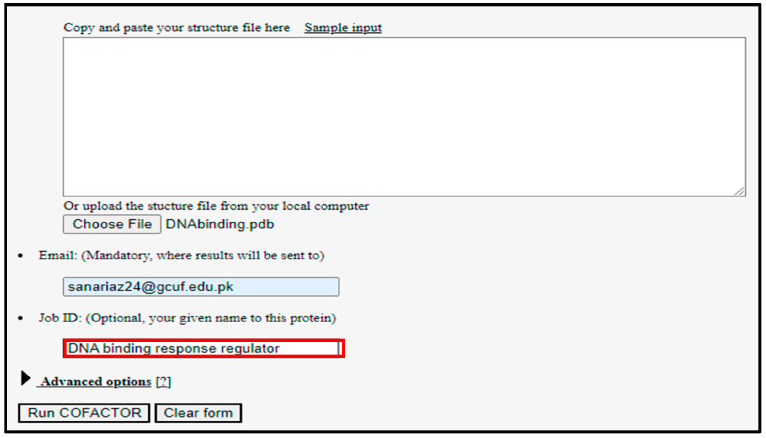
Prediction of Active sites of the protein by utilizing co factor tool for molecular docking analysis.

For the preparation of ligands, the 2D conformation of all the phytochemicals and FDA approved drugs were downloaded from PubChem [26] and the zinc database [27], to examine the potential repressing effect on the targeted proteins. In accordance with the finding of the literature review, the plant-based phytochemical and FDA-approved drugs were selected for the purpose of docking. The phytochemical sterols and alkaloids were the most frequently selected [28]. For the prediction of Active sites, Cofactor tool was used as shown in Figure 2 so that ligands can bind to that specific position and alter its function [29].

**Figure 3:**
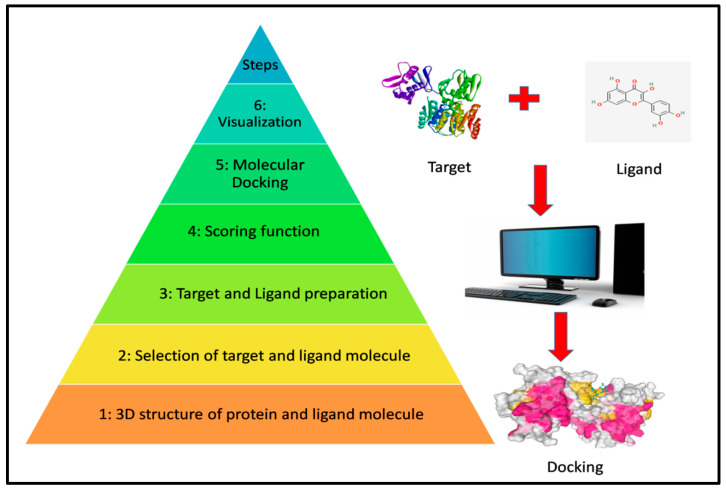
Flowchart showing the overall steps involved in protein-multiple-ligand docking for *Streptococcus pneumoniae* strain D39.

The virtual screening docking is a method that is used to cut down the price and time involved in designing and finding a drug against drug targets [30]. The workflow as shown in Figure 3 demonstrated all steps involved in protein-multiple-ligand docking. To make the protein into a stable structure in order to remove the bad torsion angle, energy minimization was carried out by using chimera software so that the ligand can bind stably with the receptor molecule as well as to remove steric clashes [31]. It allows for the identification of interaction that keep ligands attached to their respective proteins. The docking was performed by using Pyrex software 0.8 version with the Vina Wizard tool to find out the attraction between the drug targets and ligand and Biovia Discovery studio software 4.5 version was applied for the visualization of the docking pose. The structures of all the drug targets were downloaded from the PDB database by writing their 4-letter code on the search bar and saving the file in the pdb format. The preparation of all the drug targets was performed by using Biovia Discovery Studio software; all the water molecules, co-ligands, heteroatoms were eliminated and hydrogen bonds were added from the crystal structure of proteins [32]. For docking purposes, Pyrex software was used with Vina wizard tools to understand the attraction between ligand and receptor. For the preparation of macromolecules (Drug targets) all the .pdb files were converted into .pdbqt file format by clicking on the right button of the protein name and then clicking on “make macro-molecule” after loading the molecules on Pyrex.

For the preparation of ligands, all the ligands were uploaded one by one and energy minimization of total ligands was performed by right clicking on ligands and minimizing the energy and conversation of all the ligands into the .pdbqt format. All the ligands and drug target molecules were selected for docking purposes and the same procedure was performed for the remaining targets. The grid box was set based upon the active site prediction for the docking purpose. The grid box will allow you to select the search space (part of the protein, where we want to perform docking, typically called as binding site) in protein and then clicked on forward for docking. For the visualization, Biovia discovery studio and UCSF chimera were used.

### 2.11. Druglikness and Toxicity Prediction

Swiss ADME [33] and the ProTox-II tool [34] were used to evaluate potential drug-likeness qualities that are considered a critical phase in the development of the drug discovery process and should not be overlooked. Physical and chemical parameters such as molecular weight, a hydrogen bond donor, hydrogen bond acceptor, and bioavailability score were checked with the help of the Swiss ADME tool. Moreover, for the selected compound ProTox-II tool was utilized to predict toxicity. For the prediction of pharmacokinetic properties, the ADMETlab2.0 server was used.

### 2.12. MD Simulation

MD Simulation of selected complexes were conducted at 100 ns using Desmond software version 2.4 of the Schrodinger suit. The protein-ligand complex resulting from molecular docking were used in conduction MDS to obtain the dynamic interaction of the protein–ligand complex. Complexes were preprocessed, followed by optimization and minimization. OPLS_2005 forcefield was used in the minimization process. A system builder tool was used to add a transferable intermolecular potential with 3 points (TIP3P) solvent model with an orthorhombic box (10 × 10 × 10 Å). The neutralization of the model was conducted by the addition of counter ions when needed and 0.15 M of NaCl salt was included to mimic the physiological state. The conditions of NPT ensemble were 1 atm pressure and 300 K temperature. The complex was relaxed before the simulation was initiated. The trajectories were saved every 50 ps for the analysis of simulation results.

### 2.13. Binding Free Energy Calculations

MMGBSA is an empirical equation-based method that is widely used to calculate binding free energies. This method is more accurate than many molecular docking scoring functions. Hou’s team evaluated the performance of MM-GBSA approach by calculating protein–ligand binding affinities using various simulation protocols. According to their findings, the MM-GBSA method has demonstrated the ability to produce accurate results for drug design and other related research fields. Hence, the MMGBSA approach was used to calculate the binding free energies of the PDEs complexes by using the following equation:

$${\Delta G}_{\mathrm{bind}}={\;\Delta G}_{\mathrm{comp}}-{\Delta G}_{\mathrm{pro}}-{\Delta G}_{\mathrm{lig}}={\;\Delta E}_{\mathrm{ele}}+{\Delta E}_{\mathrm{vdW}}+{\Delta G}_{\mathrm{gb}}+{\Delta G}_{\mathrm{nonpol}}-\;T\Delta S$$

The ΔG_comp_, ΔG_pro_, and ΔG_lig_ show the binding energies of PDE complexes. The ΔE_ele and_ ΔE_vdW_ indicate the electrostatic and van der Waals interactions of the co-crystal ligands to PDE. The term ΔG_gb_ represents the polar solvation energy which is solved by using the Generalized Born (GB) model while ΔG_nonpol_ shows the nonpolar free-energy terms. Lastly, TΔS indicates the entropy caused by the ligands.

## 3. Results

**Figure 4:**
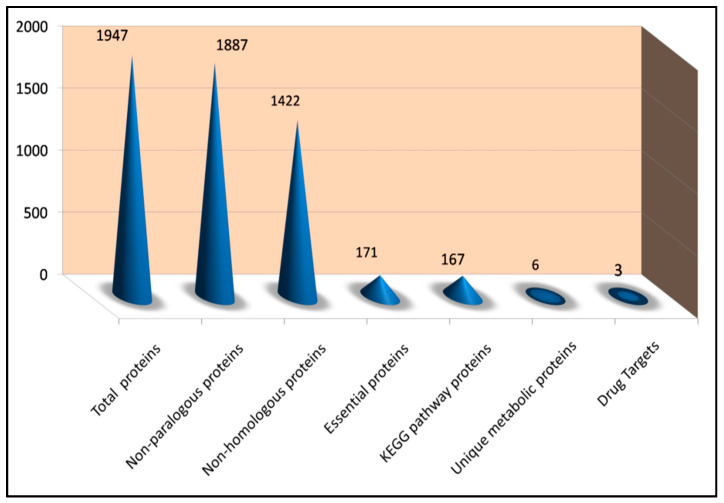
Indicating Number of proteins following the completion of each phase of the Subtractive Proteomics Approach.

To uncover and find new favorable targets against *S. pneumoniae* D39, we employed a subtractive proteomics technique. This approach has been highlighted in a variety of peer-reviewed journals for the detection, classification, and recognition of unique potential targets in human diseases [35]. Figure 4 illustrates the number of protein sequences obtained in each phase of the subtractive proteomics approach.

### 3.1. Detection of Non-Paralogous Sequences

The entire proteome of the *S. pneumoniae* D39 strain was downloaded from UniProtKB in FASTA canonical format. The proteome dataset contained a total of 1947 protein sequences. The primary purpose of this research is to identify pathogen essential, non-homologous unique proteins. This would aid in the identification of therapeutic targets for a specific strain. Following the proteome retrieval, it was required to eliminate paralogous sequences from the pathogen’s genome in order to improve the performance of other non-redundant sequences. For this purpose, the CD-HIT tool was applied. CD-HIT produced 1887 proteins from a total of 1947 proteins. The pathogen’s proteome file had 60 paralogous sequences that were eliminated.

### 3.2. Identification of Non-Homologous Protein Sequences

There is a possibility that the proteins of the pathogen and host are homologous. Hence, it is essential to locate and remove these hosts’ identical protein sequences from the pathogen proteome so that host cell toxicity is limited [36]. For this purpose, Blastp was applied with an expectation value of 0.001. After assembling the Blastp results, we discovered 1423 non-homologous sequences.

### 3.3. Identification of Essential Proteins

Essential proteins play a substantial role in the development and growth of the pathogen [37]. Essential proteins are thought to be the most promising and safe targets for drug development. For this purpose, DEG and JBrowse were used to identify the pathogen’s essential proteins that play a remarkable role in the survival and existence of this pathogen. A total of 171 essential proteins were recognized.

### 3.4. Analysis of Metabolic Pathways

**Figure 5:**
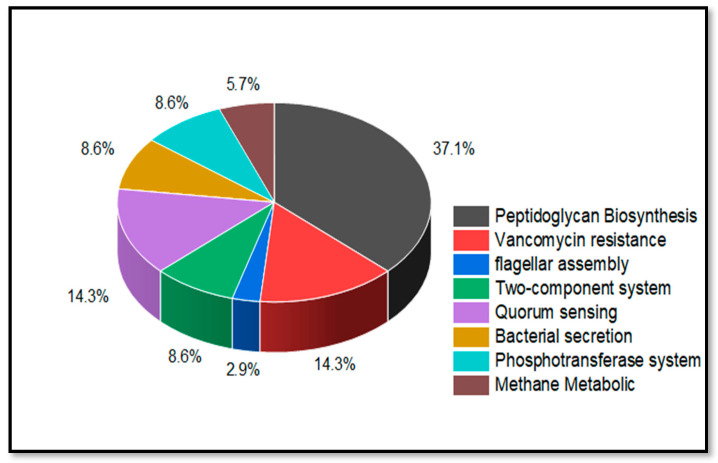
Representation of Essential Non-homologous Proteins’ Key Role in Different Metabolic Pathways.

The KEGG Pathway Database was then utilized to look for 171 essential proteins retrieved from DEG and JBrowse. Figure 5 depicts the essential roles of these proteins in pathogenic pathways. It is worth noting that 96 proteins were those with no hits in the KEGG pathway analysis. However, we found 75 proteins involved in different metabolic pathways.

The result revealed that 25 proteins out of 75 were involved in unique metabolic pathways presented in Table 1. In detail, 13 essential proteins were found as a part of Peptidoglycan biosynthesis, 5 proteins were involved in Vancomycin resistance and Quorum sensing, 1 was involved in flagellar assembly, 3 proteins were involved in the bacterial secretion system, and 2 in the component system and PhosphoTransferase system (PTS), respectively, whereas 2 proteins were involved in Methane Metabolism. From that, only six proteins were observed to be unique in *S. pneumoniae* D39 that do not share any pathway with humans and were chosen for further studies.

The distinctive pathway analysis was promising and beneficial in identifying viable and safest feasible therapeutic targets because it comprised pathogen-specific pathways.

### 3.5. Analysis and Exploration of Localization Results

The protein must be effective within the living cell for proper and consistent function. This phase is important since the drug needs to bind to a target to work, and the target present in the cell eventually helps to create a suitable drug molecule [38].

The results obtained from PSORTb and CELLO show that 83% of proteins were in the cytoplasmic region of the cell and 17% of proteins were found to be in plasma membrane proteins. Furthermore, for functional family categorization estimation of 23 essential hypothetical proteins, the Interproscan tool was utilized. The results obtained from Interproscan displayed that the most usual families include phaphorylases Gtf1, lipid kinase Glycosyl transferase, RNA-binding protein, Degv proteins, and rRNAsmall subunit methyl tarsferase1.

### 3.6. Drug Ability Analysis

In this final phase, the Drug Bank Database was used to identify and analyze druggable proteins from the *S. pneumonia* D39 strain [39]. As a result, the six finalists, unique, essential, and non-homologous protein names, were examined in the Drug Bank Database individually in order to determine their drug possibility. Table 2 indicates all the information related to drug targets obtained from the drug bank database.

It demonstrated the identification of three proteins that could be beneficial as drug targets, and they were incorporated to show similarities with the previously approved drug targets in the Drug Bank database [40]. All these three druggable proteins were located to be present in cytoplasmic region of the cell, whereas only 1 protein was present in the plasma membrane, which was discarded. As far as we know, cytoplasmic proteins are preferred as therapeutic targets.

### 3.7. Analysis of Structure Prediction and Validation

Homology modeling is considered the best-known approach in the era of drug discovery; with improvements in a machine learning techniques, it is now possible to generate a high accuracy model even from a low-identity template [41]. Homology modeling was performed to select the best templates [42]. All the information related to the quality and refinement of a structure is shown in Table 3. For DNA binding response regulator SPD_1085, the suitable template PDB ID 4kfc was identified. It is worth mentioning that GMQE ranges from 0 to 1, and the closer to 1, the better the model is [43], so it can be used for further analysis. Likewise, for UDP-N-acetylmuramate—L-alanine ligase SPD_1349, the best template PDB ID: 7bva was selected; moreover, for RNA polymerase sigma factor SigA SPD_0958 the suitable template was PDB ID: 4lK1 was selected for the molecular docking study.

The percentage distribution shown by the Ramachandran plot revealed that the overall predicted model is of better quality and can be used for further analysis as most of the region falls under the favorable zone. The Z score predicted by the ProSA-web tool represents the overall quality assessment test that shows that the predicted structure is closely related to X-ray/NMR-derived structures.

### 3.8. Protein Multiple Ligands Docking

The molecular docking method allow us to characterize how small molecules behave in the binding site of target proteins and to better understand basic biological process by simulating the interconnection among a small molecule and a protein at the atomic level. Table 4 represents the grid dimension and active sites residue of the therapeutic drug targets. In the drug discovery toolbox, molecular docking has become a lightning rod and the most appropriate technique.

**Figure 6:**
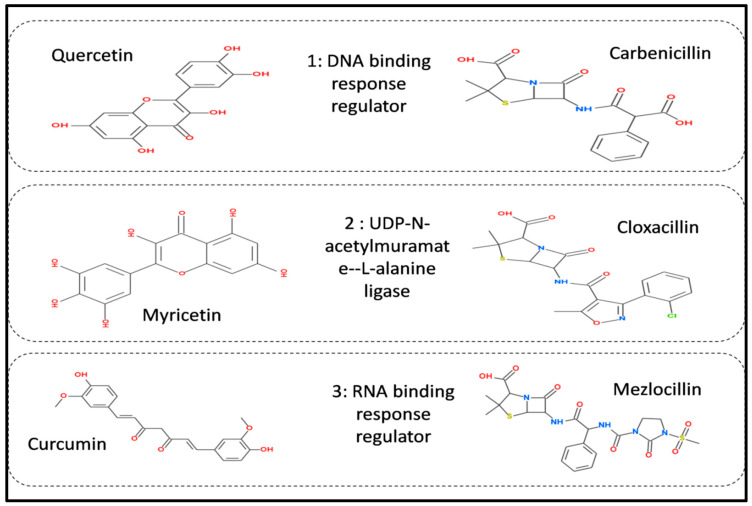
Top two ligand candidate 2D structure visualization obtained after virtual screening and further utilized for ADMET profiling.

The docking engaged two fundamental steps: outlook of the ligand structure along with its location and orientation within a pose and evaluation of the binding affinity. The results of the receptor protein structures with the phytochemical libraries and FDA approved drugs were obtained by using PyRx software. In PyRx software, the grid dimensions were set according to active site prediction obtained from co-factor tool in order to predict the protein functional sites for docking. For each drug target, the two best ligands were selected based upon the binding affinity as well as RMSD value obtained from docking results. Based on the lowest docking score value, the top two molecules from each receptor protein were chosen for further investigation as mentioned in Figure 6. Moreover, all the docked complexes were further visualized with the help of discovery studio and chimera X software version 1.4. Several types of drug interact with the protein binding sites, which are structurally and functionally crucial areas on the protein surface, to carry out the specified activity.

**Figure 7:**
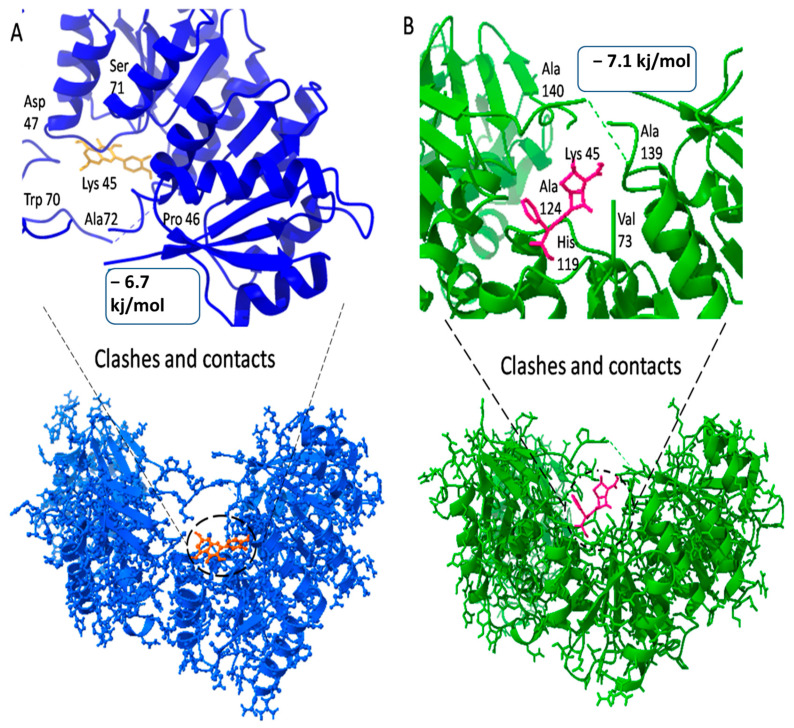
3D visualization of receptor and ligand (**A**) shows the docked complex of DNA binding response regulator with Quercetin ligand. (**B**) Represents the Carbenicillin in complex with DNA binding response regulator.

The entire compound predicted from high throughput screening with favorable binding energy was identified as a hit. The key parameter produced as a result of molecular docking is binding energy and RMSD value. Notably, the first drug target, the DNA binding response regulator (act as a receptor), showed the maximum binding affinity with the Quercetin phytochemical and Carbenicillin drug (act as a ligand) and lower root-mean-square deviation (RMSD). The binding affinity score for Quercetin phytochemical is −7.1 kj/mol, whereas the RMSD value is 1.72. Similarly, for the Carbenicillin the binding affinity score is −6.7 kj/mol, whereas the RMSD value is 1.8 as shown in Figure 7.

**Figure 8:**
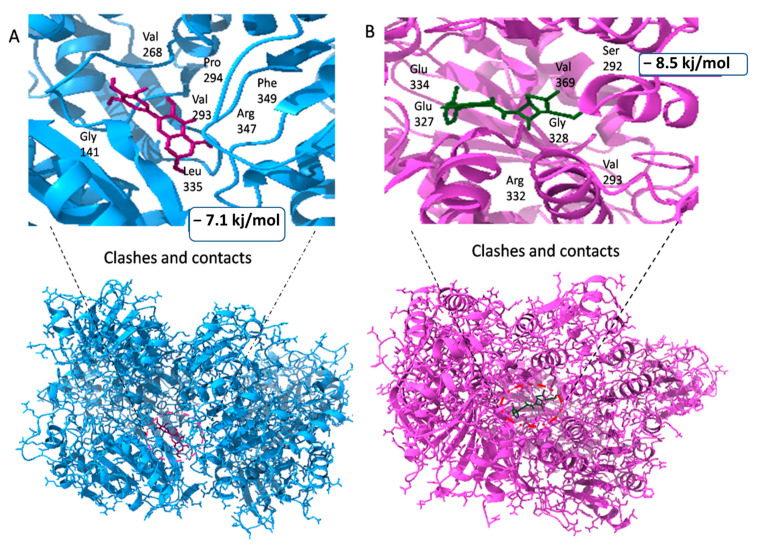
(**A**) Representation of drug target UDP-N-acetylmuramate—L-alanine ligase in docked complex with ligand Myricetin. (**B**) Shows the strong binding affinity and lowest RMSD value between UDP-N-acetylmuramate—L-alanine ligase and ligand Cloxacillin.

The second drug target that is UDP-N-acetylmuramate—L-alanine ligase (act as a receptor) shows the maximum binding affinity and lower RMSD value with Myricetin phytochemical and Cloxacillin drug (act as a ligands) as shown in Figure 8. It is noteworthy that Myricetin shows good binding affinity with the receptor protein that is −7.1 kj/mol having RMSD value is 1.63. In contrast, Cloxacillin drug shows a binding affinity of −8.5 kj/mol, having an RMSD value 1.41. Notably, the RMSD value also holds a prominent role in the process of docking. The key criteria for RMSD classification rely on two parameters. A good results shows RMSD value less than 2 Å and a favorable result occurs when the RMSD value lies between 2 Å to 3 Å [44]. Similarly, binding energy offers us a concept of the affinity and the power of interactions that occur between the ligand and the receptor molecule. It is stated that the higher the binding affinity, the less the interaction occurs, and vice versa. Thus, in the molecular docking study, we focused on the least binding energy of the compound [45].

**Figure 9:**
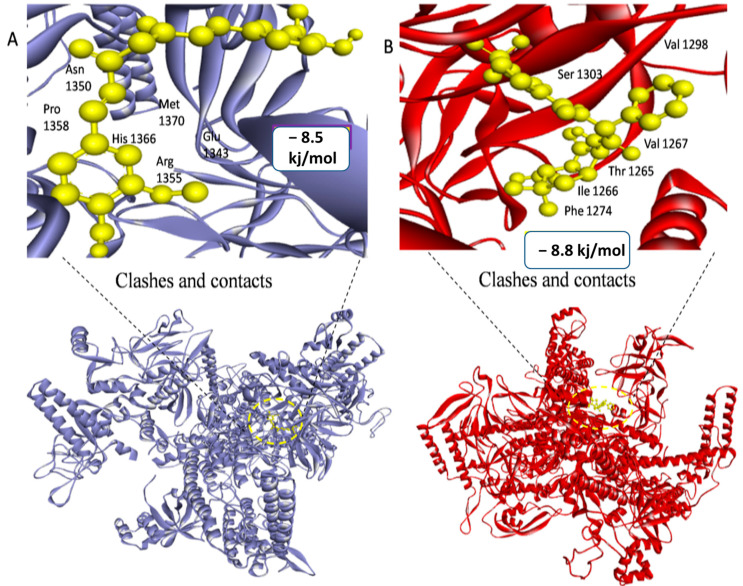
3D visualization of targeted protein with attached ligand indicating interactive residues (**A**) represent the docked complex of RNA polymerase sigma factor (act as a receptor) with Curcumin phytochemical. (**B**) Shows the good binding affinity between RNA polymerase sigma factor and Mezlocillin drug.

**Figure 10:**
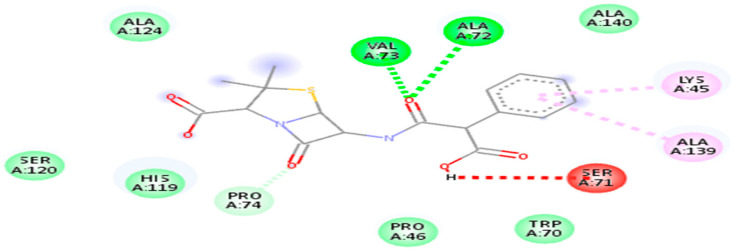
2D interaction of 4KFC with carbenicillin drug.

**Figure 11:**
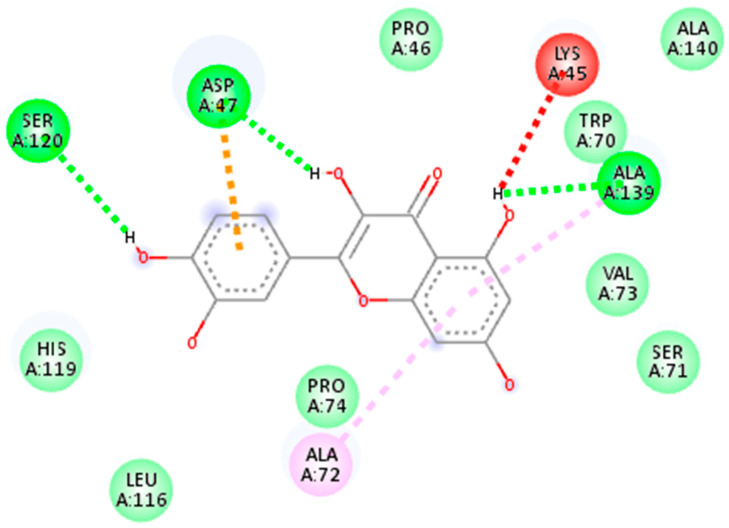
2D interaction of 4kfc with Quercetin.

**Figure 12:**
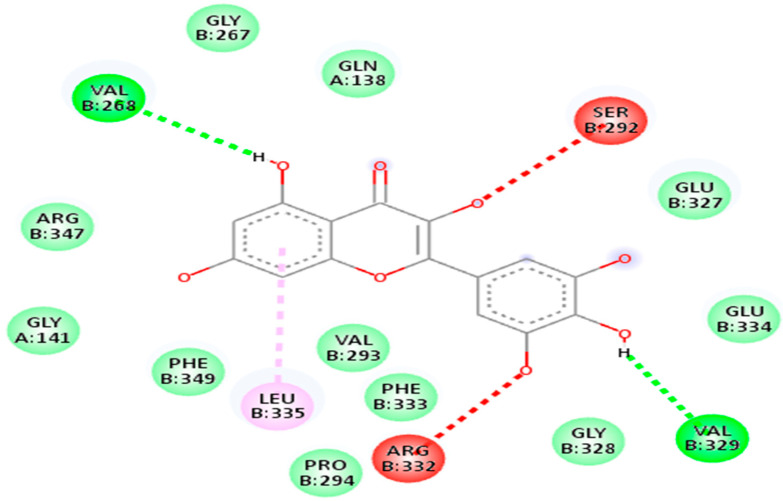
2D interaction of 7bva with Myricetin.

**Figure 13:**
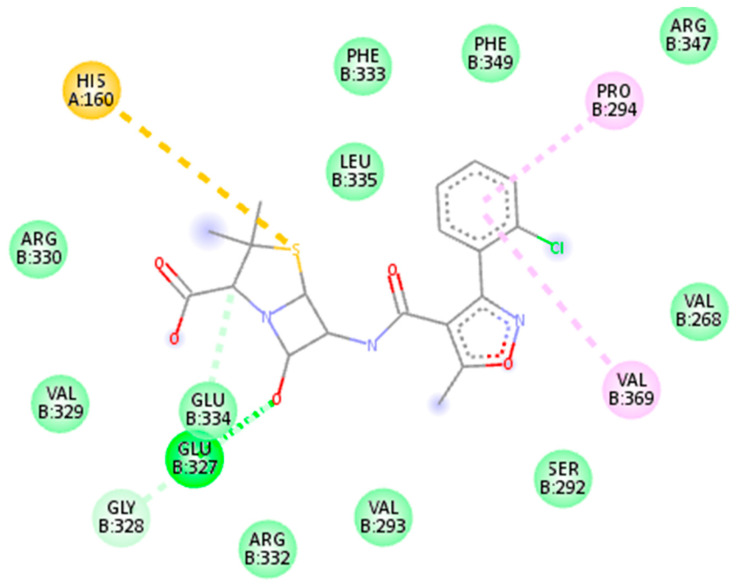
2D interaction of 7bva with cloxacillin.

**Figure 14:**
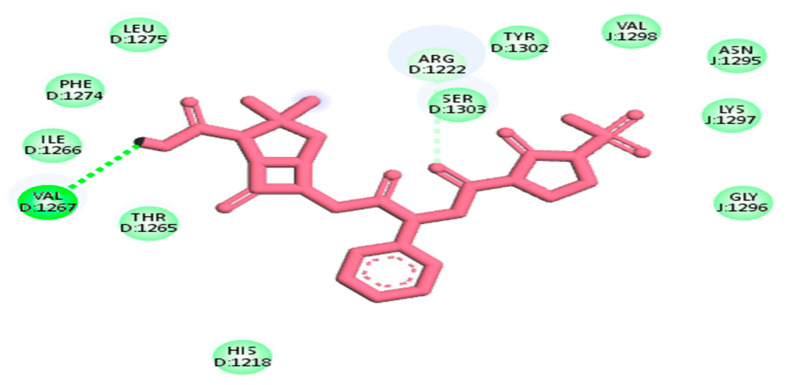
2D interaction of 4lk1 docked with Mezlocillin.

**Figure 15:**
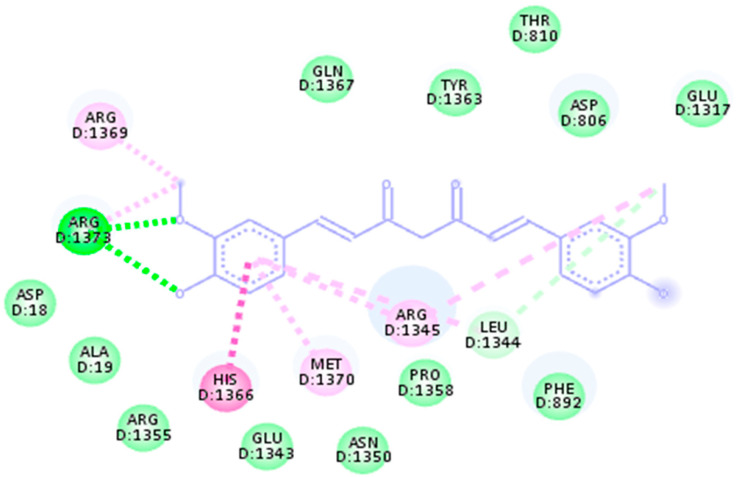
2D interaction of 4lk1 with Curcumin.

The third drug target is RNA polymerase sigma factor (act as a receptor), which shows the maximum binding affinity with the ligand Curcumin phytochemical and Mezlocillin drug (act as a ligands). Figure 9 shows the docking complex of receptor protein with the phytochemical ligand and FDA-approved drug. The binding affinity value is −8.5 kj/mol with Curcumin whereas RMSD value is 1.15. Moreover, for Mezlocillin, drug binding affinity is −8.8 kj/mol, whereas the RMSD value is 2.7. The selected compounds have strong interaction with the binding pockets of the proteins and have the lowest binding energy with the scoring function of each docked ligand. Table 5 represent the binding affinity, RMSD and interacting residues of the potent drug targets. Moreover Figure 10, Figure 11, Figure 12, Figure 13, Figure 14 and Figure 15 shows the 2D interaction of the following drug targets. All the ligands that show maximum binding energy and lower RMSD value were chosen for the ADEMET profiling and drug likeness study.

### 3.9. ADMET Profiling and Toxicity Prediction

Early in the drug development process, it is crucial to analyze the pharmacokinetic features of potential therapeutic candidates. The idea of drug-likeness serves as a helpful guide during the early stage of drug research. The drug-likeness of a compound mainly depends upon the oral bioavailability as well as the Lipinski rule of five, and indeed measures the probability that the compound will behave as an oral drug in terms of availability [33]. Table 6 represents the drug-likeness of the compounds [33].

Lipinski five rules have become the standard. It is noteworthy that the oral bioavailability score should not be less than 0.5 [46] and no more than one violation is allowed [47]. The chosen candidates had no violations of the “rule of five” and expressed drug-like properties. Moreover, the prediction of toxicity of a potent compound must be known in order to protect the human body from harmful reactions. The ADMET properties of all the ligands for the three drug targets are shown in Table 7. To identify the lead components that are active, early drug discovery depends on high-efficiency and quick ADMET profiling research. Therefore in silico toxicity prediction was carried out in order to avoid the toxic effects of the compound that could minimize the late-stage failure of the testing of the drug [48].

### 3.10. MD Simulation

**Figure 16:**
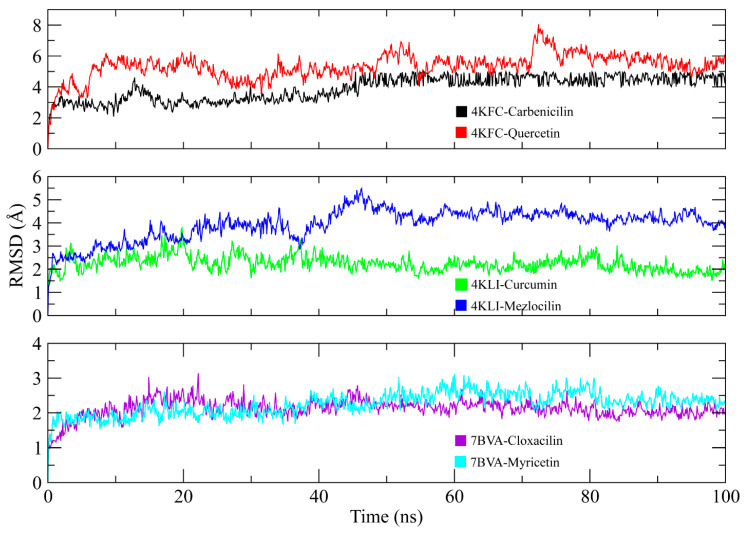
The RMSD plots of protein and ligands during 100 ns simulation.

The root-mean-square deviation (RMSD) of carbon alpha atoms of protein and ligands was calculated from the trajectories to check the stability of protein ligand complexes. It can be observed that the RMSD of 4KFC-Carbenicilin complex remained in the range of ~3 Å till 50 ns, then increased to ~4 Å and remained in this range till the end of simulation. On the other hand, the RMSD of 4KFC-Quercetin showed deviations from the start of simulation and reached to ~6 Å at 10 ns. The RMSD remained in this range till the end of the simulation, with some deviations between 70 to 75 ns. In case of 4KLI complexes, the RMSD of Curcumin complex showed stable behavior throughout the simulation in the range of ~2–3 Å, while the Mezlocillin complex gradually increased to ~5 Å at 50 ns and then attained stability in the range of ~4–4.5 Å till the end of simulation. The complexes of 7BVA showed stable behavior in terms of RMSD values in both complexes as both complexes remained in the range of ~2–3 Å throughout the simulation. The RMSD plots of all complexes are shown in Figure 16.

**Figure 17:**
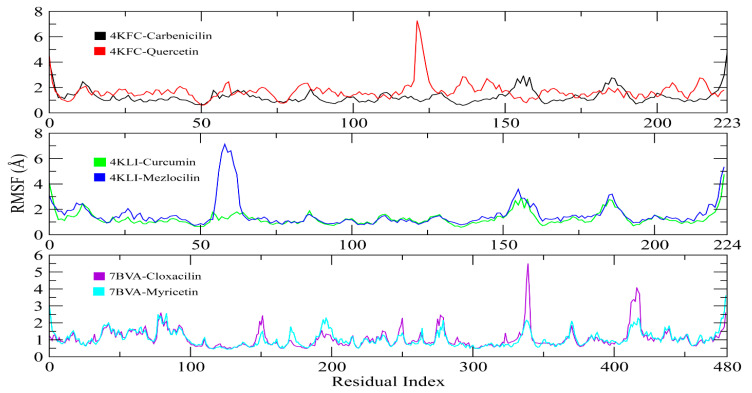
The fluctuations of protein residues during simulations as determined by RSMF values.

Root-mean-square fluctuations (RMSF) valued were calculated to determine the dynamic behavior of protein restudies when bound to these ligands. RMSF values of protein residues fluctuate less than 2 Å in the entire simulation period, except for the loop regions in all complexes (Figure 17). The RMSF figure indicated that the protein residues showed more flexibility when bound to the Carbenicillin in 4KFC-Carbenicilin compared to the 4KFC-Quercetin complex. Similarly, in the 4KLI complexes, the complex of Mezlocillin showed more fluctuations. Lastly, the 7BVA-Cloxacilin complex showed more fluctuations than the 7BVA-Myricetin complex. The overall fluctuations suggested that the protein–ligand complex was stable.

**Figure 18:**
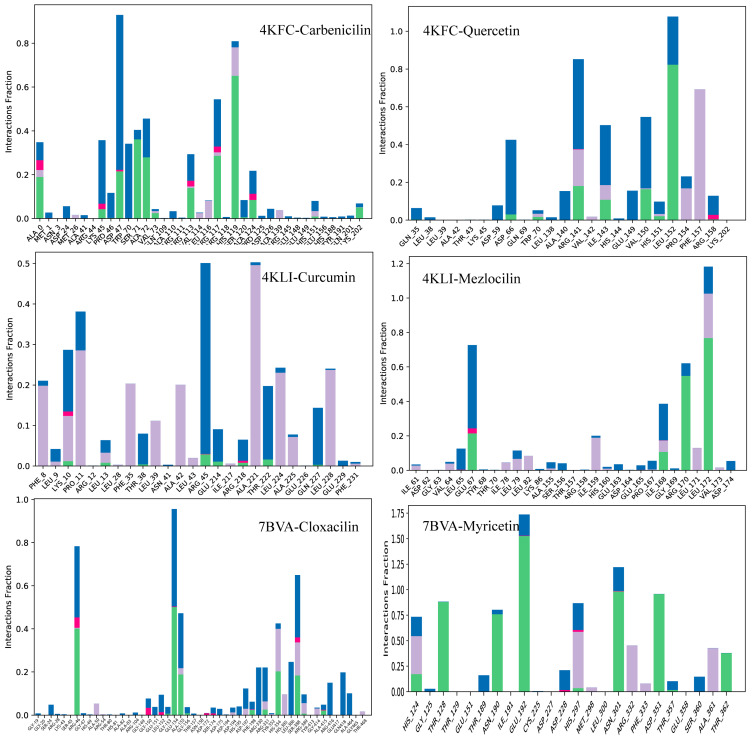
The interaction of proteins and ligands during MDS. The interacting residues are shown are shown as large, stacked bars. Green bars show hydrogen bonding, pink show ionic bonds, blue shows the water bridges, and grey show hydrophobic contacts.

The most important interactions between the protein and ligands were hydrogen bonds, ionic bonds and hydrophobic interactions determined through MDS. The residues involved in hydrogen bonding in 4KFC-Carbenicilin complex were: Ala1, Lys45, Asp47, Ser71, Ala72, Val73, Arg113, Arg117, His119, Ala124, Lys202. While Asp66, Trp70, Arg141, Ile143, Val150, His151, and Leu152 were involved in hydrogen bonding in the 4KFC-Quercetin complex. In protein–ligand contact analysis of 4KLI complexes, Lys10, Leu13, Arg45, Glu214, Arg218, and Thr222 formed hydrogen bonds with Curcumin and Mezlocilin was involved in hydrogen bonding with Glu67, Ile168, Arg170, and Leu172. Lastly, Cloxacin formed hydrogen bonds with Arg46, Glu154, Ala155, Arg330, His385, Arg389, and Arg416 while His124, Thr128, Asn190, Glu192, Asn301, Asp351, and Thr362 were involved in hydrogen bonding with Myricetin. The protein–ligand contacts of all complexes are shown in Figure 18.

### 3.11. MM/GBSA

The molecular mechanics Generalized Born surface area (MM/GBSA) method was used to calculate the total binding free energy for all complexes. MMGBSA dG Bind value is usually used to estimate the stability of the protein–ligand complex. The lower values of MMGBSA dG Bind indicates that the complex is more stable and vice versa. It was computed as a sum of the protein-ligand complex and the difference in the protein and its ligand’s free energies. The total binding free energy estimated using the MM/GBSA model is the outcome of the contribution of various protein–ligand interactions such as van der Waals energy, Covalent energy, Coulomb energy, and solvent energy. The total binding free energies of the complexes are given in Table 8.

## 4. Discussion

*Streptococcus pneumoniae* is a notorious pathogen that is responsible for the high rate of incidence of meningitis, lobar pneumonia, otitis media and bacteremia. The dramatic rise in penicillin resistance and the limited availability of the pneumococcal vaccine suggest that morbidity from pneumococcal illness may increase [49]. There is an utmost need to discover new potent drug targets. One of the most powerful approaches utilized in bioinformatics by existing research is the subtractive proteomics method for identifying and selecting pathogen-specific therapeutic targets [50]. This method is entirely in silico and employs proteome analysis to identify the key proteins of a disease without triggering any interruption, such as the disruption of the host proteome.

Although Subtractive proteomic analysis of *S pneumoniae* has already been reported in two studies, the prevalence of the D39 strain of *S. pneumoniae* was very high, and during the past ten years, it has been the primary serotype-specific cause of childhood meningitis in developing nations [9]. D39 is a historically significant serotype 2 strain that was utilized in research by Avery and colleagues to demonstrate that DNA is the genetic material of concern. Aside from its historical significance, strain D39 has become a major model of pneumococcal pathogenesis. Despite being discovered nearly a century ago, D39 remains exceedingly virulent in murine infection models and is likely the strain employed most frequently in current investigations of pneumococcal pathogenesis. Therefore, the major and chief interest of the present study was to find out new drug targets in *S. pneumoniae* strain D39. Moreover, gaps from the previous research on *S. pneumoniae*, such as the detailed study of metabolic pathways and ADMET profiling, are highlighted in this current study.

A total of 1947 proteins from the whole proteome of *S. pneumoniae* strain D39 were analyzed. In most large protein datasets, some patterns are significantly similar (paralogous sequences), and removing those sequences is important for preserving time and processing assets. For this purpose, the CD-HIT tool was used with a cut-off value of 0.8 (80%). From a total of 1947 proteins, CD-HIT generated 1887 proteins. 60 paralogous sequences from the pathogen’s proteome file were removed [18].

The survival of a cell or an individual depends on the presence of essential genes. Bacteria cannot survive if these essential proteins are degraded or modified. We can kill bacteria and cure disease by targeting these proteins. Essential proteins are preferred targets for vaccine and antibacterial drug development. Thus, 171 essential proteins were identified [51]. Notably, not all essential genes can be exploited as therapeutic drug targets because some of them may be linked to the host metabolic pathways. Therefore, manual comparison between the host and the pathogen was carried out by KEGG database. Out of total 171 essential proteins, only six proteins were observed to be involved in unique pathways in *S. pneumoniae* D39. The findings of the pathogen-specific pathway identification are consistent with those of *L. interrogans*, *A. baumannii*, and *S. saprophyticus* published by Amineni U et al., Goyal et al. and Shahid et al.

Subcellular localization prediction is a quick and very affordable approach to determining the function of a certain protein. Furthermore, it was shown that proteins can localize at multiple places; hence, localization is an important part of designing any therapeutic agent. Because proteins positioned on the membrane are difficult to purify and study, cytoplasmic proteins are thought to be more appropriate as therapeutic targets.

The drug screening action would be immensely assisted by collective homology to previously identified drug targets, helping in rational curative discovery and repositioning. As a result, all the essential non-homologues proteins that were involved in the unique pathways of the bacteria were compared to the DrugBank database by performing BLASTp. The result of the DrugBank showed three druggable target proteins, namely DNA binding response regulator, UDP-N-acetylmuramate—L-alanine ligase and RNA polymerase sigma factor as therapeutic targets.

3D structures of these target proteins were predicted by Swiss model through a homology modeling approach. Plant-based phytochemicals and FDA-approved drugs were chosen for docking. Molecular docking analysis is a fundamental and crucial technique for drug discovery. It enables the prediction of molecular interactions that hold a protein and a ligand together in the bound state. Molecular docking is a technique for predicting a ligand’s affinity, orientation, and interaction in a protein binding site. In docking the selected compounds have strong interaction with the binding pockets of the proteins and have the lowest binding energy with the scoring function of each docked ligand. All the RMSD values are less than 3. All the ligands that show maximum binding energy and lower RMSD value were chosen for the ADEMET profiling and drug likeness study. It is noteworthy, that the drug target DNA binding response regulator shows the docked complex with Quercetin phytochemical which is indeed a polyhydroxy flavonoid derivative occurring in plants’ flowers, leaves, and fruits, and it has a variety of pharmacological actions [52]. It considerably lowered the ratio of wet/dry weight of lung tissue in animal studies, alleviating inflammation and pathological damage to lung tissue as well as providing efficient and detailed protection from *S. pneumoniae* infection. Similarly, the second drug target UDP-N-acetylmuramate—L-alanine ligase shows maximum binding affinity with Myricetin phytochemical. Myricetin has previously been identified as a promising anti-inflammation, anti-tumor, antifungal, antimicrobial, and anti-virulence natural compound [53].

A powerful drug candidate is quickly absorbed and evenly spread across the human body to ensure efficient metabolism and effect. Notably, rejection of pharmaceutical drugs during clinical studies due to adverse side effects because of their toxicity appears to be very costly for drug development. Therefore, ADMET profiling is carried out that is considered as the critical phase in any drug discovery process. This was accomplished through the SWISSADME database, which revealed that certain drugs have favorable pharmacokinetic features. The Lipinski rule of five was employed to estimate the drug like properties and molecular characteristic of the selected complex. Surprisingly, all the compounds fulfil the drug likeness criteria.

Animal testing has been used almost exclusively as a technique of determining the hazardousness of substances. At least tens of thousands of animals must be sacrificed in order to obtain all of the toxicological results for a single drug utilizing laboratory animals. To overcome this hurdle all the docked complexes were further analyzed for the toxicity prediction and ADME analysis. Surprisingly, none of the compounds show violation in BBB, whereas two compounds show low GI absorption. Moreover, metabolism contributes a crucial role in the evaluation of the drug and drug-drug cross reaction. Cytochrome sub families includes (CYP1A2, CYP3A4, CYP2D6, CYP2C9, and CYP2C19) are responsible for the drug metabolism [54]. Most of the compounds revealed no drug–drug cross reactivity. Furthermore, none of the compound shows the toxicity except Curcumin which shows a slightly toxic effect but could be modified into a non-toxic type during the lead optimization stage of the drug discovery [55].

This fact revealed that mentioned compounds can be used for the discovery of potent drugs in the safest manner. Moreover, the result of ADMET analysis revealed that certain drugs have favorable pharmacokinetic properties. As a result, the extracted phytochemical can be exploited as effective therapeutic candidates against *S. Pneumoniae* since they can limit the function of proteins involved in the disease by targeting their binding pockets.

## 5. Conclusions

Antimicrobial resistance is rapidly spreading, prompting researchers to explore novel therapeutic targets that could aid in the development of antibacterial drugs. The current work has found three novel targets in the *Streptococcus pneumoniae* D39 strain because certain targets are involved in pathogenic-specific metabolic pathways and were effectively addressed in other bacteria, the current study investigated developing medicines against these targets. Consequently, this study represents a significant step forward with the discovery of novel anti *S. pneumoniae* compounds. Further in vivo/in vitro, research should investigate these targets to see if they affect the lifetime and pathogenicity of *S. pneumoniae*.

## Figures and Tables

**Table 1:** Non-homologous crucial proteins engaged in unique metabolic pathways obtained from KEGG database.

Protein Name	Unique Pathways	Common Pathways
Bifunctional protein GlmU	Spd 00541 O-antigen nucleotide sugar biosynthesis	Spd 01250 Biosynthesis of nucleotide and Spd 00520 Amino sugar and nucleotide sugar metabolism.
RNA polymerase sigma factor	Spd 02040 Flagellar assembly	
UDP-N-acetylmuramoyl-L-alanyl-D-glutamate—L-lysine ligase	Spd 00550 Peptidoglycan biosynthesis	Spd 01100 Metabolic pathway
Single-stranded DNA-binding protein	Spd00550 Peptidoglycan biosynthesis	Spd03030 DNA replication, Spd03430 Mismatch repair, and Spd03440 Homologous recombination
Phospho-N-acetylmuramoyl-pentapeptide-transferase	Spd 01502 Vancomycin resistance and Spd00550 Peptidoglycan biosynthesis	Spd 01100 Metabolic pathway
Protein translocase subunit SecA	Spd 02024 Quorum sensing, Spd 03070 Bacterial secretion system and Spd 01110 Biosynthesis of secondary metabolites	Spd 03060 Protein export
Glycerol-3-phosphate Acyltransferase	Spd 01110 Biosynthesis of secondary metabolites	Spd 00561 Glycerolipid metabolism and Spd 00564 Glycerophospholipid metabolism
UDP-N-acetylmuramoyl-tripeptide—D-alanyl-D-alanine ligase	Spd 01502 Vancomycin resistance, Spd 00550 Peptidoglycan biosynthesis and Spd 00300 Lysine biosynthesis	Spd 01100 Metabolic pathway
DNA-binding response regulator	Spd 02020 Two-component system	
PTS system, IIB component, putative	Spd02060 PhosphoTransferase system (PTS)	
Capsular polysaccharide biosynthesis protein	Spd00541 O-antigen nucleotide sugar biosynthesis Spd00552 Teichoic acid biosynthesis	Spd01250 Biosynthesis of nucleotide sugars Spd01100 Metabolic pathways
UDP-N-acetylmuramate—L-alanine ligase	Spd 00550 Peptidoglycan biosynthesis	
Transcriptional regulator ComX1 (Transcriptional regulator ComX2)	Spd 02020 Two-component system Spd02024 Quorum sensing	
Acyl carrier protein (ACP)	Spd 01110 Biosynthesis of secondary metabolites,	Spd 01100 Metabolic pathways
Penicillin-binding protein 2B	Spd 00550 Peptidoglycan biosynthesis Spd 01501 beta-Lactam resistance	
Nitroreductase family protein	Spd 00541 O-antigen nucleotide sugar biosynthesis	Spd 01100 Metabolic pathways
Preprotein translocase, SecE subunit	Spd 02024 Quorum sensing Spd 03070 Bacterial secretion system	Spd 03060 Protein export
DNA polymerase III, delta subunit (EC 2.7.7.7)		Spd 03030 DNA replication, Spd 03430 Mismatch repair, and Spd 03440 Homologous recombination
Replicative DNA helicase (EC 3.6.4.12)		Spd 03030 DNA replication
Phosphomevalonate kinase (EC 2.7.4.2)	Spd 01110 Biosynthesis of secondary metabolites	Spd 00900 Terpenoid backbone biosynthesis
Lipid II isoglutaminyl synthase	Spd 00550 Peptidoglycan biosynthesis	Spd 00110 metabolic pathway
Acyltransferase domain protein	Spd 01110 Biosynthesis of secondary metabolites	Spd 00561Glycerolipid metabolism Spd 00564 Glycerophospholipid metabolism
Alanine racemase (EC 5.1.1.1)	Spd 01502 Vancomycin resistance	Spd 00470 D-Amino acid metabolism and Spd 00110 metabolic pathway
D-alanine—D-alanine ligase	Spd01502 Vancomycin resistance and Spd00550 Peptidoglycan biosynthesis	Spd01100 metabolic pathway
Isopentenyl-diphosphate delta-isomerase	Spd01110Biosynthesis of secondary metabolites	Spd00900Terpenoid backbone biosynthesis

**Table 2:** Information about 3 drug targets of *S. pneumoniae* D39 Obtained from Drug Bank Database.

S.R Num	Protein Name	Gene Name	Uniprot ID	Drug Bank ID	Location
1	DNA-binding response regulator	SPD_1085	Q9A515	DB01972	Cytoplasm
2	UDP-N-acetylmuramate—L-alanine ligase	murC SPD_1349	P45066	DB01673	Cytoplasm
3	RNA polymerase sigma factor SigA	rpoD SigA SPD_0958	Q18BX5	DB08874	Cytoplasm

**Table 3:** Information about the quality and refinement of a structure by using saves v6.0.

Proteins Name	ERRAT Quality Factor	VERIFY 3D Compatibility Score	PROCHECK Ramachandran	ProsA-Web
DNA binding response regulator	92.82%	96.87% compatibility score	Core 89.2%	−7.27
			Allowed 10.0%	
			General 0.5%	
			Disallowed 0.3%	
UDP-N-acetylmuramate—L-alanine ligase	95.92%	97.91% compatibility score	Core 93.2%	−12.31
			Allowed 6.6%	
			General 0.1%	
			Disallowed 0.0%	
RNA polymerase sigma factor	81.18%	66.16% compatibility factor	Core 88.7%	−6.02
			Allowed 10.1%	
			General 0.1%	
			Disallowed 0.3%	

**Table 4:** Representation of grid dimension as well as active sites residue for three potent Drug targets.

Drug Targets PDB ID	Dimension Angstrom X	Dimension Angstrom Y	Dimension Angstrom Z	Active Site Residues Obtained from Co-Factor Tool
4KFC	29.474	35.94	39.61	Leu21, Ser35, Arg27, Leu34, Gln35, Leu38, Ala41, Lys45, Pro46, Trp70, Ser71 and Ala72
7BVA	29.66	25	28.42	Lys126, Thr121, Thr123, His124, Gly125, Lys126, Thr128, Ser131, Ile134, Val135, Gly141, Glu334, Glu327, Ser292 and His160
4LK1	149.36	152.76	157.24	Ala42, Arg44, Ile46, leu48, Ser50, Pro52, Gly151, Ser178, Glu181, Ile183, Val185, Asn208, Thr210, Ile217, Arg219, Ala221, leu224, His1366, Met1370, Arg1355, Glu1343, Asn1350 and Pro1358

**Table 5:** The most promising candidates’ binding affinity, RMSD and interacting residues.

**1: DNA Binding Response Regulator**				
**Compound ID**	**Compound Name**	**Binding Affinity**	**RMSD**	**Interacting Residues**
20824	Carbenicillin	−6.7 kj/mol	1.8	Lys45, pro46, Trp70, Ser71, Ala72, Val73, pro74, His119, Ser120, Ala 124 and Ala 139
5280343	Quercetin	−7.1 kj/mol	1.72	Lys45, Pro46, Asp47, Trp70, Ser71, Ala72, Val73, Pro74, Leu116, His119, Ser120, Ala139 and Ala140
**2: UDP-N-acetylmuramate—L-alanine ligase**				
5281672	Myricetin	−7.1 kj/mol	1.63	Arg332, Pro294, Phe333, Val293, Leu335, Phe349, Gly141, Arg347, Val268, Gly267, Gln138, Ser292, Glu327, Glu334, Val329 and Gly328
6098	Cloxacillin	−8.5 kj/mol	1.41	Glu334, Glu327, Val329, Gly328, Arg332, Val293, Ser292, Val369, Val268, Arg330, His160, Leu335, Phe333, Phe349, Pro294 and Arg347
**3: RNA Polymerase Sigma Factor**				
969516	Curcumin	−8.5 kj/mol	1.15	His1366, Met1370, Arg1355, Glu1343, Asn1350, Pro1358, Leu1344, Phe892, Asp18, Ala19, Arg1373, Arg1369, Gln1367, Tyr1363, Thr810, Asp806 and Glu1317
656511	Mezlocillin	−8.8 kj/mol	2.7	Val1267, Thr1265, Ile1266, Phe1274, Leu1275, His1218, Arg1222, Ser1303, Tyr1302, Val1298, Asn1295, Lys1297 and Gly1296

**Table 6:** Drug-likeness properties of potential compounds.

**1: DNA Binding Response Regulator**				
**Compound Ids**	**Molecular Weight**	**Hydrogen Bond Donor**	**Hydrogen Bond Acceptor**	**Oral Bio-Availability Score**
5280343	302.24	5	7	0.55
20824	378.4	3	6	0.56
**2: UDP-N-acetylmuramate—L-alanine ligase**				
5281672	318.24	5	8	0.55
6098	435.88	2	6	0.56
**3: RNA Polymerase Sigma Factor**				
969516	368.38	2	6	0.55
656511	499.5	3	8	0.55

**Table 7:** Potential compounds ADMET profiling of top drug candidates.

Target Proteins						
Parameters	1: DNA Binding Response Regulator		2: UDP-N-acetylmuramate—L-alanine ligase		3: RNA Polymerase Sigma Factor	
	ID 5280343	ID 20824	ID 5281672	ID 6098	ID 969516	ID 656511
Absorption/Distribution						
Blood-Brain Barrier	No	No	No	No	No	No
GI Absorption	High	Low	Low	High	High	High
p-gp substrate	No	Yes	No	Yes	No	Yes
Caco-2 permeability	−5.20	−6.15	−5.65	−5.38	−4.83	−6.073
Metabolism						
CYP1A2 inhibitor	Yes	No	Yes	No	No	No
CYP2C19 inhibitor	No	No	No	Yes	No	No
CYP2C9 inhibitor	No	No	No	No	Yes	No
CYP2D6 inhibitor	Yes	No	No	No	No	No
CYP3A4 inhibitor	Yes	No	Yes	Yes	Yes	No
Toxicity						
Cytotoxicity	In active	In active	In active	In active	In active	In active
Immuno-toxicity	In active	In active	In active	In active	In active	In active
AMES toxicity	Non-toxic	Non-toxic	Non-toxic	Non-toxic	Non-toxic	Non-toxic
Rat oral acute toxicity	Non-toxic	Non-toxic	Non-toxic	Non-toxic	Non-toxic	Non-toxic

**Table 8:** The Binding free energies of the complexes calculated by implying prime-MM/GBSA.

Target	Compound	MMGBSA dG Bind	MMGBSA dG Bind Coulomb	MMGBSA dG Bind Covalent	MMGBSA dG Bind Solv GB	MMGBSA dG Bind vdW
4KFC	Carbenicilin	−0.04115	−0.5658100	−0.0147365	0.56198971	−0.0389674
4KFC	Quercetin	0.064942	−0.6541846	0.0019109	0.73494300	−0.0163880
4KLI	Curcumin	−5.46 × 10^−12^	3.64 × 10^−12^	0	−9.55 × 10^−12^	−1.14 × 10^−13^
4KLI	Mezlocilin	−2.18 × 10^−11^	8.19 × 10^−12^	−4.55 × 10^−13^	−2.80 × 10^−11^	−1.14 × 10^−13^
7BVA	Cloxacilin	−18.7583	42.80804	6.310527	−6.20121	−41.8799
7BVA	Myricetin	−30.5268	−4.07858	4.33391	6.111314	−17.6194

## Data Availability

Not applicable.
